# Designing Gel-Inspired Food-Grade O/W Pickering Emulsions with Bacterial Nanocellulose–Chitosan Complexes

**DOI:** 10.3390/gels11080577

**Published:** 2025-07-24

**Authors:** Antiopi Vardaxi, Eftychios Apostolidis, Ioanna G. Mandala, Stergios Pispas, Aristeidis Papagiannopoulos, Erminta Tsouko

**Affiliations:** 1 Theoretical and Physical Chemistry Institute, National Hellenic Research Foundation, 48 Vassileos Constantinou Avenue, 11635 Athens, Greecepispas@eie.gr (S.P.); apapagiannopoulos@eie.gr (A.P.); 2 Laboratory of Food Process Engineering, Department of Food Science & Human Nutrition, Agricultural University of Athens, Iera Odos 75, 11855 Athens, Greeceimandala@aua.gr (I.G.M.); 3 Division of Genetics & Biotechnology, Department of Biology, National and Kapodistrian University of Athens, Zografou Campus, 15784 Athens, Greece

**Keywords:** eco-friendly emulsions, gel-inspired, polymer complexes, biobased emulsifier

## Abstract

This study explored the potential of chitosan (CH)/bacterial cellulose (BC) complexes (0.5% *w*/*v*) as novel emulsifiers to stabilize oil-in-water (o/w) Pickering emulsions (20% *v*/*v* sunflower oil), with a focus on their gel-like behavior. Emulsions were prepared using CH combined with BNC derived via H_2_SO_4_ (BNC1) or H_2_SO_4_-HCl (BNC2) hydrolysis. Increasing BNC content improved stability by reducing phase separation and enhancing viscosity, while CH contributed interfacial activity and electrostatic stabilization. CH/BNC1_25:75_ emulsions showed the highest stability, maintaining an emulsion stability index (ESI) of up to 100% after 3 days, with minimal change in droplet size (R_h_ ~8.5–8.8 μm) and a positive ζ-potential (15.1–29.8 mV), as confirmed by dynamic/electrophoretic light scattering. pH adjustment to 4 and 10 had little effect on their ESI, while ionic strength studies showed that 0.1 M NaCl caused only a slight increase in droplet size combined with the highest ζ-potential (−35.2 mV). Higher salt concentrations led to coalescence and disruption of their gel-like structure. Rheological analysis of CH/BNC1_25:75_ emulsions revealed shear-thinning behavior and dominant elastic properties (G′ > G″), indicating a soft gel network. Incorporating sunflower-seed protein isolates into CH/BNC1 (25:75) emulsions led to coacervate formation (three-layer system), characterized by a decrease in droplet size and an increase in ζ-potential (up to 32.8 mV) over 7 days. These findings highlight CH/BNC complexes as sustainable stabilizers for food-grade Pickering emulsions, supporting the development of biopolymer-based emulsifiers aligned with bioeconomy principles.

## 1. Introduction

Emulsions play a crucial role in various industries, including food, pharmaceuticals, and cosmetics, where their stability directly influences product quality and performance. Due to their thermodynamically unstable nature, emulsions eventually undergo phase separation via mechanisms such as coalescence, creaming, sedimentation, flocculation, or Ostwald ripening. To mitigate this instability, gel-forming thickening agents can be incorporated to increase the viscosity of the aqueous phase [1]. Additionally, amphiphilic emulsifiers such as proteins, polymers, and surfactants reduce interfacial tension, thereby enhancing kinetic stability [2,3]. However, some emulsifiers fail to provide long-term stability, and in addition, certain synthetic low-molecular-weight surfactants may pose risks to human health or the environment [4]. With growing consumer awareness on healthier lifestyles and sustainability, the demand for eco-friendly, food-grade emulsifiers has risen over the past decade [5]. As a result, there is an increasing need to replace synthetic surfactants with natural gel-compatible emulsifiers [6,7].

In this context, particle-stabilized emulsions, known as Pickering emulsions, have attracted interest in the food and pharmaceutical industries due to their natural surface-active properties and robust stabilization mechanisms [2]. Various carbohydrate polymers such as cellulose, chitosan (CH), chitin, and starch, along with salt-soluble globulins, water-soluble albumins, and plant proteins, are frequently utilized as colloidal particles to stabilize Pickering emulsions [8,9,10,11]. Among these, bacterial cellulose (BC) and CH have gained significant attention due to their intrinsic gel-forming abilities and functional versatility, presenting a promising avenue for environmentally friendly and biocompatible emulsification systems.

BC is an extracellular homopolysaccharide produced by *Acetobacter* species using various carbon resources, renewable or of commercial grade. Due to high surface density of hydroxyl groups, BC exhibits pronounced hydrophilicity, facilitating strong interactions with water molecules. Additionally, its amphiphilic nature, crystalline structure, and extensive hydrogen bonding confer stability and order [12,13]. BC can be ex-situ modified through acid (H_2_SO_4_ or HCl or acid mixtures) or enzymatic (cellulase) hydrolysis, yielding nanostructures with enhanced properties such as a large specific surface area, superior mechanical strength, high crystallinity, and biodegradability [14]. The amphiphilic characteristics of BC and its derivatives make them ideal candidates for Pickering emulsification, eliminating the need for synthetic surfactants. The combination of hydrophilic hydroxyl groups and hydrophobic interactions from the crystalline polymer network allows BC to adsorb at the oil-water interface, effectively stabilizing the emulsion. Notably, BC has been reported to inhibit droplet aggregation across a broad temperature range when utilized in oil-in-water (o/w) Pickering emulsions by Zhai XiChuan et al., 2018 [15]. Although BC-based emulsions have been shown to be biodegradable, non-toxic, and biocompatible [16], there is limited information on the specific application of ex-situ modified BC as stabilizers in food-grade emulsions. Further exploration on the stabilization mechanisms of BC in such applications, particularly in the context of food systems, is required [17]. CH is a cationic polysaccharide derived from the deacetylation of chitin and is known for its biodegradability, non-toxicity, and biocompatibility [18]. However, its low solubility in neutral aqueous environments limits its direct application in Pickering emulsions. To overcome this, various chemical modifications have been developed to improve its emulsifying functionality, including the synthesis of CH derivatives or conjugates with negatively charged macromolecules [19]. More specifically, CH exhibits strong electrostatic attraction to the negatively charged BNC, promoting the formation of stable interfacial complexes between these two biopolymers. By physically blending BNC and CH, fully bio-based synergistic systems could be created that effectively stabilize emulsions without relying on conventional synthetic surfactants. The inherent negative charge of BNC, combined with the positive charge of CH, leads to the formation of gel-like networks composed of polyelectrolyte complexes, which significantly enhance emulsification capacity and colloidal stability. These modifications not only improve CH solubility but also facilitate its interfacial activity and gel-forming potential, enabling its use in structured emulsion gels. This strategy enhances emulsion robustness and offers promising opportunities for multifunctional applications, including targeted drug delivery and food-grade emulsion stabilization, aligning with the growing demand for sustainable and biocompatible materials [20].

In this study, we developed and evaluated novel biopolymer-based systems to stabilize oil-in-water (o/w) Pickering emulsions containing 20% (*v*/*v*) sunflower oil. Responding to the growing demand for eco-friendly, and food-grade emulsifiers as alternatives to synthetic counterparts, we focused on natural, gel-compatible biopolymers—specifically CH and BNC. Emulsions stabilized by individual CH, BC, and BC derived via H_2_SO_4_ (BNC1) and H_2_SO_4_–HCl (BNC2) were first assessed. Building on this, CH was systematically combined with BNC1 and BNC2 to form hybrid CH/BNC complexes at varying ratios (25:75, 50:50, 75:25), maintaining a total emulsifier concentration of 0.5% (*w*/*v*). The effect of pH and ionic strength on CH/BNC1-stabilized emulsions was examined to evaluate colloidal robustness under varying environmental conditions. As a proof of concept, this study investigated the ability of CH/BNC1 (25:75) complexes to encapsulate sunflower seed (SFS) protein isolates (PI). Emulsions were characterized over 7 days under ambient conditions, using visual inspection, dynamic/electrophoretic light scattering (DLS/ELS), polarized light microscopy, and rheological analysis. The novelty of this work lies in the design and characterization of CH/BNC nanocomplexes with dual functionality—as both stabilizers and nanocarriers—enabling targeted delivery and enhanced formulation versatility. This study presents a novel strategy to produce sustainable, soft gel-like nanocomplexes that serve as eco-friendly emulsifier systems, offering a versatile platform for Pickering emulsions with promising applications in functional food and nutraceutical formulations.

## 2. Results and Discussion

### 2.1. Evaluation of Parent O/W Emulsions

**Figure 1:**
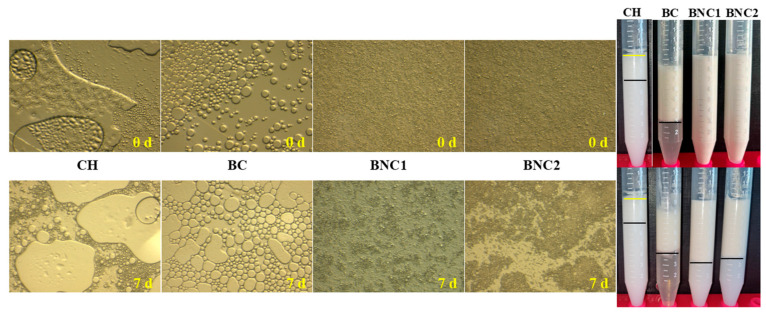
Microscopic and visual evaluation of parent o/w Pickering emulsions (20 *v*/*v* sunflower oil and 0.5 wt. total emulsifier) using (from left to right) neat chitosan (CH), bacterial cellulose (BC), BC after H_2_SO_4_ treatment (BNC1) and BC after H_2_SO_4_-HCl treatment (BNC2) as stabilization agents, at the day of preparation (0 d) and after 7 days of storage at ambient temperature. The left panel presents polarized light microscopy micrographs, providing insights into structural stability phenomena. The right panel shows macroscopic images of the emulsions, highlighting potential phase separation over time.

**Figure 2:**
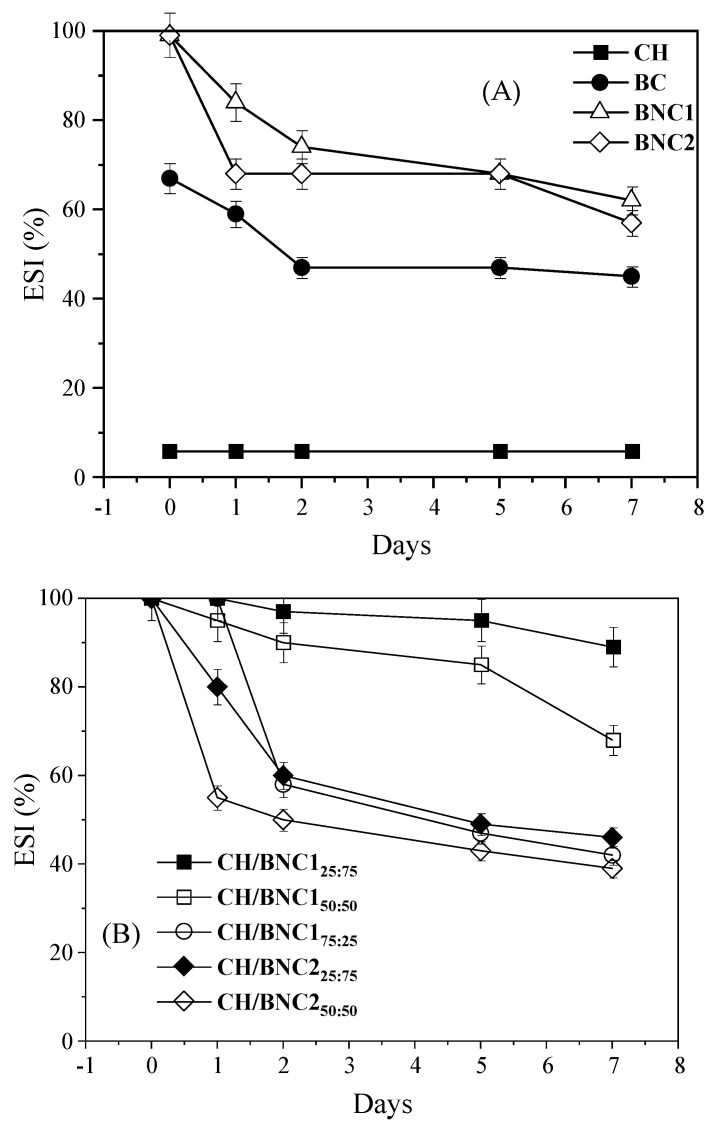
Emulsifying stability index (ESI) of (**A**) parent o/w Pickering emulsions (20 *v*/*v* sunflower oil and 0.5 wt. total emulsifier) stabilized with neat chitosan (CH), bacterial cellulose (BC), BC after H_2_SO_4_ treatment (BNC1), and BC after H_2_SO_4_-HCl treatment (BNC2) and (**B**) complex emulsions using complexes of CH/BNC1_25:75_, CH/BNC1_50:50_, CH/BNC1_75:25_, CH/BNC2_25:75_, CH/BNC2_50:50_, CH/BNC2_75:25_ as stabilizers. The ESI was assessed at specified intervals to evaluate the emulsions’ resistance to phase separation and structural integrity over time, at ambient temperature. Data are presented as mean ± standard deviation from two independent experimental replicates.

O/w emulsions stabilized by CH, BC, and BC obtained from H_2_SO_4_- and H_2_SO_4_-HCl-assisted hydrolysis (BNC1 and BNC2, respectively) were initially prepared and investigated (Figure 1 and Figure 2A, Table 1). The CH-stabilized emulsions exhibited intensive phase separation immediately after preparation, with an ESI of 6%. The instability was attributed to the protonation of amine groups (−NH_3_^+^) in CH, which increased hydrophilicity at pH < pKa of the polycationic polysaccharide, limiting the formation of aggregates capable of stabilizing oil droplets [5]. As a result, oiling-off and significant coalescence were observed. The ζ-potential of CH emulsions ranged from 4.9 to 10.7 mV, indicating weak electrostatic repulsion. Despite an increase in ζ-potential over storage, the droplet size also increased from 134 nm to 211 nm, with the formation of large aggregates up to 7.5–8.1 μm. This behavior is consistent with findings of Ahmed et al., 2021 [21], where CH emulsions in acidic conditions exhibited instability, whereas emulsions prepared at pH 6.0 showed a rather improved emulsification capacity with smaller droplet sizes (~0.8 μm)

BC-stabilized emulsions exhibited higher initial stability compared to CH, with an ESI of 67% at 0 days (Figure 2A). However, significant phase separation was observed over time, with the ESI decreasing to 45% after 7 days. The droplet size followed a bimodal distribution, varying from 147 nm–7.8 μm (size range at 0 d) to 260 nm–7.6 μm (size range at 7 d). The ζ-potential values increased (in absolute values) over storage. Notably, after 7 days, a creamy layer was formed at the top of the falcon, yet with a clean yellowish serum layer at the bottom, indicating thus the dispersion of droplets with different sizes and densities [22]. Paximada et al., 2016 [17] reported similar behavior for olive oil/BC emulsions (serum index of 3% and droplet size of d_3,4_ = 26 μm), which exhibited stronger interfacial interactions compared to SO/BC emulsions (this study), potentially explaining the increased phase separation observed in this study.

Pickering o/w emulsions stabilized by BNC1 and BNC2 revealed excellent emulsifying capacity (ESI: 99% for both emulsions) at 0 h (Figure 2A). Both emulsions exhibited polydisperse droplet distributions, with BNC1 forming droplets of 81 nm, 192 nm, and 8 μm, while BNC2 formed droplets of 89 nm, 156 nm, and 8 μm (Table 1). The phase separation in both emulsions was evident after 7 days of storage with ESI declining to 62% for BNC1 o/w emulsions and 57% for BNC2-based ones (Figure 2A). For both emulsifiers, optically opaque emulsions with turbid serum at the bottom were observed, rather than a clear serum, signifying that flocculation phenomenon occurred. In such systems, flocculated droplets cream to form an upper opaque layer, while non-flocculated droplets disperse in the lower phase [22]. The droplet size distribution increased significantly upon storage in both cases. These results align with findings of Sommer & Staroszczyk, 2023 [23] who demonstrated that BC nanocrystals obtained after H_2_SO_4_-assisted hydrolysis provided higher emulsion stability under stress conditions (e.g., sonication and ultracentrifugation), but still showed phase separation after prolonged storage (3-d). Similarly, emulsions (1% *v*/*v* SO) stabilized with BC nanofibrils (prepared by TEMPO-mediated oxidation) formed larger droplets compared to those stabilized with nanocrystals (after 60% *w*/*w* H_2_SO_4_ hydrolysis), as nanofibrils exhibit a higher aspect ratio, slowing their adsorption at the oil-water interface [24,25].

The negative surface charge of both BNC emulsions followed an upward tendency upon storage. High surface charges on nanocellulose can cause electrostatic repulsion with the negatively charged oil-water interface (due to OH^-^ absorption), leading to a weakly adsorbed layer around oil droplets. This results in coalesced droplets with reduced surface area yet increased size and problems in maintaining stable emulsions at low nanocellulose concentrations [26]. Therefore, the high surface charge detected on the 7th day, accompanied by the increased average droplet size and declined ESI for both emulsions, justified their instability over time. Overall, BNC-stabilized emulsions demonstrated better emulsification capacity than CH and BC, making both stabilizers promising candidates for further investigation.

**Figure 3:**
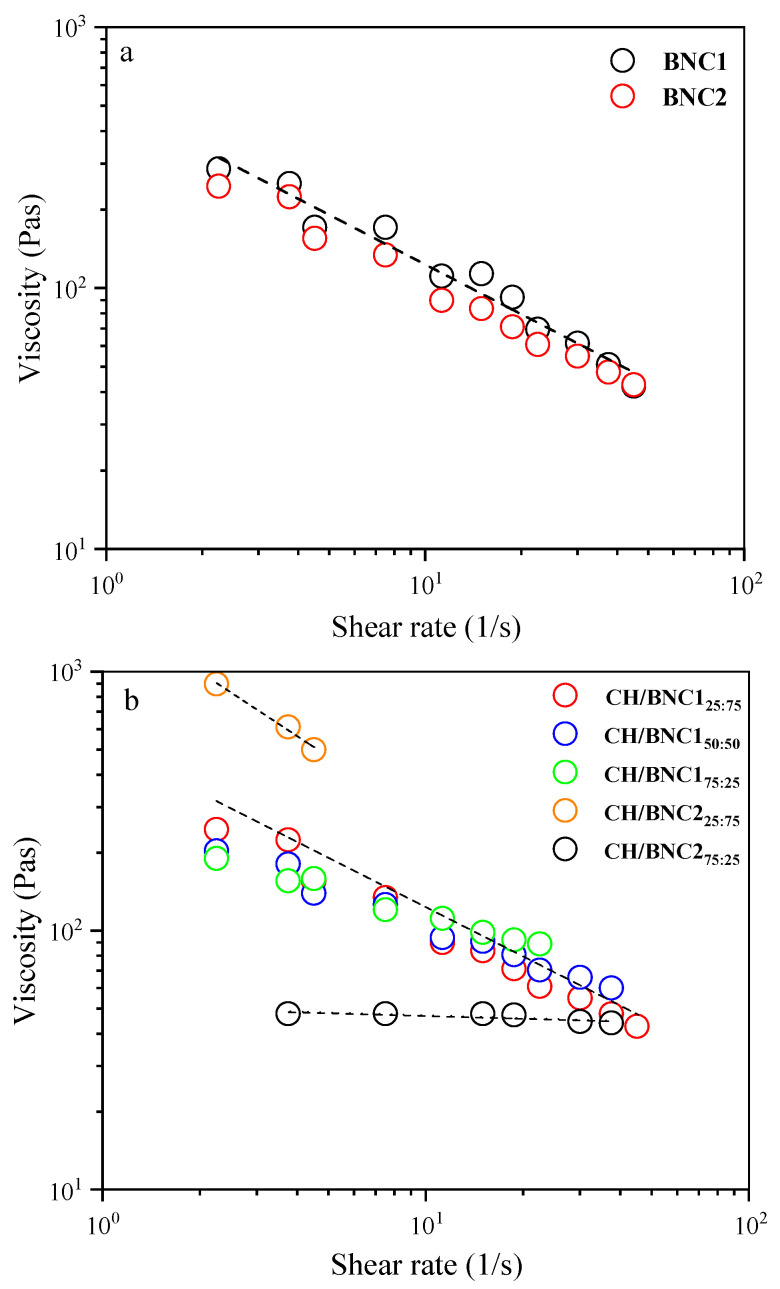

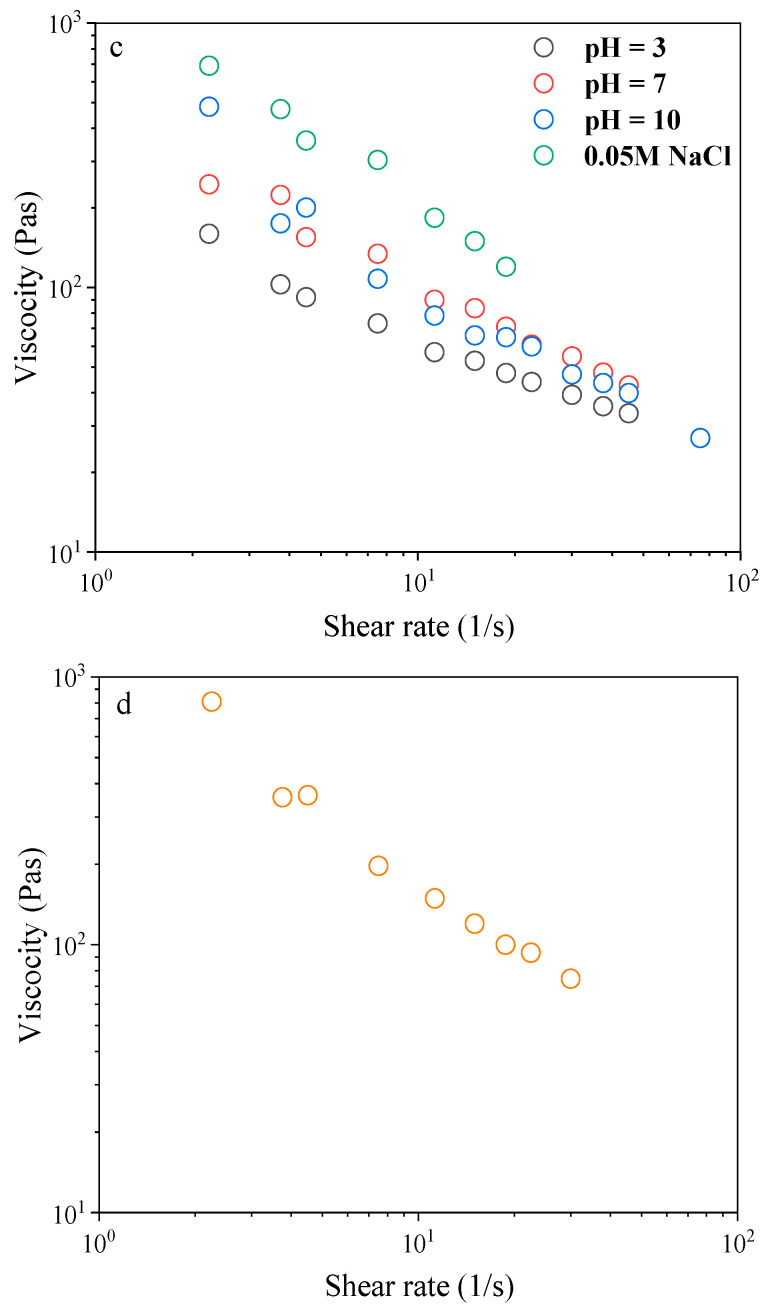
Shear-thinning behavior under stimuli of (**a**) parent o/w Pickering emulsions (20 *v*/*v* sunflower oil and 0.5 wt. total emulsifier) stabilized with bacterial cellulose (BC) after H_2_SO_4_ treatment (BNC1), and BC after H_2_SO_4_-HCl treatment (BNC2), (**b**) complex emulsions using complexes of CH/BNC1_25:75_, CH/BNC1_50:50_, CH/BNC1_75:25_, CH/BNC2_25:75_, CH/BNC2_50:50_, CH/BNC2_75:25_ as stabilizers, (**c**) complex emulsions using CH/BNC1_25:75_, under various pH values (3–10) and 0.05 M NaCl (ionic strength), and (**d**) complex emulsions using CH/BNC1_25:75_, combined with protein isolates extracted from sunflower seed after 7 d of storage at ambient temperature. Dashed lines are examples of typical fits with Equation (3). (power-law model).

Regarding CH o/w and BC o/w emulsions (Figure 3), viscosity measurements were unreliable. It can be deduced that the closed-packed arrangement of droplets on the 7th day required high centrifugal force to compress them to an open state or could have led to erratic flow behavior [22]. This was also evident in the polarized microscope images (Figure 1), where the large droplets in the CH-stabilized emulsions portrayed the oiling-off phenomenon. The coalescence phenomenon might be defined as the huge droplets that formed from the merger of smaller ones and subsequently were grouped around droplets of varying sizes in the BC-based emulsions [22]. Paximada et al., 2016 [17] depicted micrographs of olive oil-BC and olive oil-CMC o/w emulsions with similar droplet polydispersity and after ultrasonication treatments.

On the contrary, the shear-thinning behavior observed for BNC-based emulsions indicated a structured network that deformed under shear. As the shear rate progressively rose, the shear force destroyed the emulsion’s droplet network, which was reflected in a decline in viscosity (Figure 3). Previous studies reported a similar rheological tendency for TEMPO-oxidized bacterial cellulose-stabilized emulsions [27]. The non-Newtonian behavior also aligned with the visual inspection using polarized microscopy. Figure 1 displays BNCs microparticle coverage on the droplet surface interaction, upon which a rather heterogenous distribution with closed-arranged droplets emerged from the BNC1 emulsifier utilization rather than BNC2.

### 2.2. Characterization of CH/BNC Complexes

Given the superior emulsification capacity of BNCs over neat CH and BC, CH/BNC complexes at different ratios were investigated for their stabilizing efficiency (Table 1). Since the self-assembly behavior of CH/BNC complexes in water plays a crucial role in shaping droplet morphology and interfacial dynamics, understanding these interactions could provide new strategies for enhancing emulsion stability. By fine-tuning the ratio and interactions between these biopolymers, the possibility to develop more stable, long-lasting emulsions with greater resistance to coalescence and phase separation was explored. To further evaluate these effects, the assembly behavior of CH/BNC complexes in water was analyzed. It was observed that the average particle size of CH/BNC complexes increased significantly with the addition of CH (*p* < 0.05). Specifically, CH/BNC1 complexes exhibited particle size distributions ranging from 111 nm to 1149 nm for a 25:75 ratio, reaching up to 244 nm–2426 nm for a 75:25 ratio. Similarly, CH/BNC2 complexes formed aggregates between 78 nm and 856 nm at a 25:75 ratio and between 38 nm, 222 nm, and 2021 nm at a 75:25 ratio. Multi-modal size distributions were obtained for neat BNC dispersions. Neat BNC1 dispersions exhibited aggregate sizes of 74 nm and 471 nm, while BNC2 dispersions showed aggregates of 60 nm, 387 nm, and 2969 nm. On the other hand, the complete dissolution of CH in an acidic environment (pH < pKa 6.5) [28] led to the formation of chitosan coiled-like structures of 84 nm in size.

While neat BNCs tend to flocculate in aqueous suspensions, the incorporation of CH, a polyelectrolyte, improved the dissolution stability of CH/BNC complexes, especially when the complexation occurs in alkaline environments (Karppinen et al., 2012 [29]). However, the physical blending of CH and BNC presents limitations, including potential phase separation or precipitation due to the intrinsic chemical differences between the polymers [30]. This observation aligns with the polydispersity index (PDI) results in this study, where high PDI values (~0.5) were detected for all CH/BNC complexes, indicating phase separation upon complexation.

The electrophoretic mobility of CH/BNC complexes followed an upward positive trend as the CH content increased. For CH/BNC1 complexes, the ζ-potential values were determined as 47.6 mV (25:75), 53.4 mV (50:50), and 62.5 mV (75:25), while for CH/BNC2, values were equal to 32.9 mV (25:75), 38.1 mV (50:50), and 48.3 mV (75:25). The ζ-potential of CH alone was approximately 54.1 mV, whereas BNC1 and BNC2 exhibited values of −34.7 mV and −27.5 mV, respectively (Table 1). The electrostatic interactions between the amino groups of CH and sulfate groups of BNC1 and sulfate and carboxyl groups of BNC2 created macro-ion complexes with positive surface charges and stability, which is attributed to the CH addition. It has been reported that the ζ-potential of microcrystalline cellulose/CH complexes was 24.5 mV, with higher molecular weight CH leading to an increased ζ-potential of 64.1 mV [31]. Conversely, the assembly of carboxymethyl CH and BC resulted in negatively charged complexes (−35.2 mV at pH 7.0) due to repulsive electrostatic interactions [32].

### 2.3. Characterization of Pickering O/W Complex Emulsions

**Figure 4:**
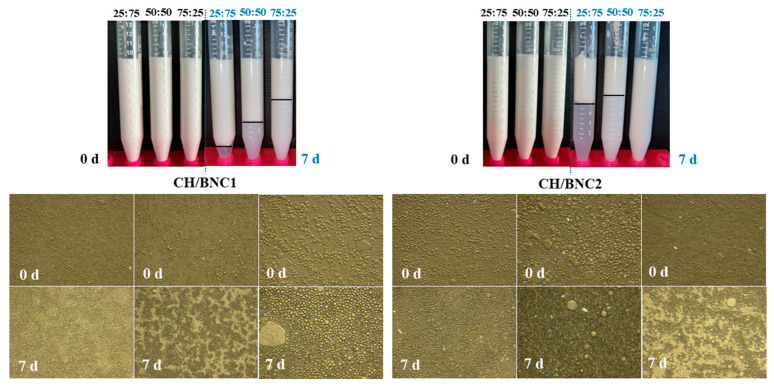
Visual (**top panel**) and microscopic evaluation (**bottom panel**) of complex o/w Pickering emulsions (20 *v*/*v* sunflower oil and 0.5 wt. total emulsifier) using complexes of chitosan (CH), BC after H_2_SO_4_ treatment (BNC1) and BC after H_2_SO_4_ -HCl treatment (BNC2) at various ratios (CH/BNC1_25:75_, CH/BNC1_50:50_, CH/BNC1_75:25_, CH/BNC2_25:75_, CH/BNC2_50:50_, CH/BNC2_75:25_), at the day of preparation (0 d) and after 7 d of storage at ambient temperature.

Next, the produced CH/BNCs complexations were explored as a potential strategy to stabilize o/w emulsions. Emulsification was carried out using CH/BNC complexes at different ratios (25:75, 50:50, 75:25), maintaining a constant total emulsifier concentration of 0.5% wt. All the freshly prepared o/w emulsions demonstrated excellent kinetic stability (ESI: 99%) (Figure 2B) without any indication of phase inversion (Figure 4). However, upon storage under ambient conditions, instability mechanisms such as creaming, sedimentation, and flocculation emerged, varying in intensity among the different formulations. The CH/BNC1 (25:75) emulsions exhibited exceptional stability during the first three days of storage (ESI: 100%), with moderate gravitational separation observed on day 7, where droplet migration led to the formation of a creamy top layer and a transparent bottom phase (ESI: 89%). At the 25:75 ratio (CH/BNC1), the BNC content dominated, which was correlated with strong adsorption at the oil-water interface due to its amphiphilic nature and high surface area. CH, though less abundant, contributed to film-forming properties, creating thus a robust interfacial layer that delayed gravitational separation (e.g., coalescence or sedimentation). CH/BNC1 (50:50) and CH/BNC1 (75:25) emulsions underwent creaming at different rates, forming two-layer systems with distinct opacity, reflecting variations in droplet concentration. The ESI of CH/BNC1 (50:50) decreased to 68%, while CH/BNC1 (75:25) dropped to 42% after 7 d. In contrast, emulsions prepared using CH/BNC2 at all ratios exhibited pronounced gravitational separation. CH/BNC2 (25:75) and CH/BNC2 (50:50) emulsions primarily underwent creaming, while CH/BNC2 (75:25) emulsions exhibited sedimentation. Notably, in CH/BNC2 (75:25) emulsions, sedimentation was accompanied by oiling-off on day 7, attributed to prolonged droplet coalescence. This instability was evident as early as day 1, with the dispersed and continuous phases remaining highly immiscible, making ESI estimation unreliable (data not included in Figure 2B). CH/BNC2 (25:75) and CH/BNC2 (50:50) emulsions displayed a droplet-rich cream layer at the top, while the serum phase exhibited differences in lightness due to the presence or absence of concentrated small droplets. Their gravitational instability was further confirmed by the decline in ESI to 50% after 7-d of storage.

The droplet size distributions of the o/w Pickering emulsions were visually inspected, along with their movement over time. DLS studies revealed multi-modal size distributions with sizes ranging from nanoscale to macroscale in all the prepared emulsions, accompanied by high PDI values during storage (0.29–0.52) (Table 1). In heterogeneous CH/BNC1 (25:75) emulsions, macroscale droplets decreased in size (R_h_ = 8.4 μm to R_h_ = 7.8 μm), while nanoscale droplets (from R_h_ = 75 nm, 133 nm to R_h_ = 228 nm) increased, suggesting droplet fragmentation. Conversely, CH/BNC1 (50:50) and CH/BNC1 (75:25) emulsions exhibited coalescence of nanosized droplets and partial collapse of microscale ones (Table 1), which was reflected in the turbidity variations of the serum layers. Similar trends were observed in CH/BNC2 (25:75) and CH/BNC2 (50:50) emulsions, where microsized droplets aggregated progressively over 7 d of storage. Nonetheless, CH/BNC2 (75:25) emulsions exhibited extensive breakdown, with a marked decrease in droplet sizes (R_h_ = 90 nm/1 μm/8.3 μm at 0 day to R_h_ = 56 nm/1.4 μm/8.0 μm at 7 d), supporting the observed coalescence and subsequent sedimentation during storage [33].

The ζ-potential of all emulsions exhibited an increasing trend over time (Table 1). CH/BNC1 (25:75) and CH/BNC2 (25:75) emulsions had similar initial surface charge values (5.88 mV and 5.17 mV, respectively), indicating that the dominant BNC content diluted the positive charge contribution from CH. The CH/BNC1 (50:50) ratio exhibited intermediate ζ-potential values (11.7 mV) at 0 d, whereas CH/BNC2 (50:50) showed a slightly negative ζ-potential (−1.26 mV), suggesting a balance between the positive charge of CH and the negative charge of BNCs. The type of acid treatment applied to BC influenced the overall charge distribution in the emulsifier complex. The highest positive ζ-potential values were observed in CH/BNC1 (75:25) and CH/BNC2 (75:25) emulsions, where the higher CH content allowed its positive charge to dominate. These results suggest that emulsion droplets maintained sufficient electrostatic repulsion to prevent aggregation. A ζ-potential exceeding 30 mV is typically required to ensure emulsion stability, preventing droplet aggregation; lower values indicate weaker repulsive forces, leading to flocculation and phase separation [15]. In this study, ζ-potential values remained below 30 mV, particularly for CH/BNC2 emulsions, supporting the instability observed after 7 d of storage. Similar findings were reported by Ahsan et al. (2020) [31], where neutralized charge distributions were observed in microcrystalline cellulose/CH emulsions at pH 7.0. Razavi et al. (2020) [34], also prepared unstable bacterial nanocellulose (after H_2_SO_4_-assisted hydrolysis)/fish gelatin o/w emulsions (with cinnamon essential oil), exhibiting low surface charges (4–12 mV) after 30 d of storage at 42 °C.

Overall, emulsion stability was strongly dependent on BNC concentration. At high BNC concentrations, CH/BNC-based emulsions demonstrated improved stability, whereas lower BNC concentrations resulted in poorer emulsification performance. Increased BNC content facilitated greater particle adsorption at the droplet interface, reducing droplet size and enhancing emulsion stability [35]. However, instability in certain emulsions upon storage may be attributed to entropic and enthalpic-driven attraction between colloidal particles, which accelerated creaming instability at specific polymer concentrations, promoting bridging flocculation rate [32]. A similar emulsification mechanism was reported by Zhang et al., 2022 [32] who identified a threshold polymer concentration beyond which flocculation occurred. At pH 7.0, ESI% increased as BC concentration rose from 0 wt% to 0.2 wt%, with BC/CCS dispersions exhibiting optimal emulsification properties when BC exceeded 0.1 wt%.

The overall trend for most emulsions showed a decrease in viscosity as the shear rate increased, indicating shear-thinning behavior (Figure 3b), which is typical for emulsions where the internal structure (e.g., droplet network or nanoparticle interactions) breaks down under shear, leading to lower viscosity. CH/BNC1 (25:75) and CH/BNC1 (50:50) emulsions revealed a steeper decrease in viscosity with increasing shear rate, indicating a more pronounced shear-thinning behavior. This could imply a less stable emulsion structure that breaks down more readily under shear. On the other hand, CH/BNC1 (75:25) and CH/BNC2 (75:25) had a relatively flat viscosity profile, indicating a weaker shear-thinning effect, suggesting thus a network structure that resists breakdown under shear. With respect to CH/BNC2 (25:75) emulsions, they showed a high initial viscosity suggesting a strong network, yet it appeared to abruptly break down under shear, causing a significant drop in viscosity and indicating thus a highly structured but shear-sensitive emulsion. The high heterogeneity (PDI~0.44) and the charge neutralization of CH/BNC2 (50:50) emulsions, which further reduce their ability to adsorb effectively at the oil-water interface, and the insufficient electrostatic repulsion between droplets could cause them to aggregate, forming flocs, and thus leading to unreliable results. Hence, the potential presence of flocs could significantly alter the flow behavior, which may deform, break apart, or move unpredictably under shear, leading to fluctuations in viscosity readings [22]. Increased shear-thinning behavior as BC strength increases in BC:CMC emulsions was reported by [36].

H_2_SO_4_ hydrolysis (CH/BNC1 (25:75), CH/BNC1 (50:50), CH/BNC1 (75:25)) demonstrated a decrease in both N and m with increasing CH ratio (Table 2), indicating that higher CH content leads to weaker shear-thinning behavior and lower apparent viscosity. This could be attributed to a reduced network density or weaker interactions within the emulsion. In contrast, H_2_SO_4_-HCl hydrolysis (BNC2, CH/BNC2 (25:75), and CH/BNC2 (25:75)) did not exhibit a systematic trend due to the outlier behavior of CH/BNC2 (25:75). BNC2, in the absence of CH, displayed relatively high N and m values, while CH/BNC2_25:75_ exhibited exceptionally high N (1778) and m (0.82), suggesting a more viscous and strongly shear-thinning system, potentially due to enhanced BC network formation under H_2_SO_4_-HCl hydrolysis. Conversely, CH/BNC2 (25:75) had the lowest N (51) and m (0.035), behaving as a Newtonian fluid with low viscosity. These findings highlight the critical influence of both composition and hydrolysis protocol on rheological properties, demonstrating the potential to tailor emulsion viscosity for specific applications. Overall, the shear-thinning and elastic-like behaviors observed in the CH/BNC-stabilized emulsions indicated the formation of structured networks due to effective interfacial coverage and droplet–droplet interactions. Emulsions with higher BNC content (e.g., CH/BNCI (25:75)) showed more pronounced viscoelasticity, reflecting stronger Pickering stabilization and possible physical linking of droplets. In contrast, the CH/BNCII (50:50) emulsion showed early flocculation and failed rheological measurements, likely due to charge neutralization and unstable droplet interfaces. The loss of measurable viscosity at high ionic strength further confirmed the role of electrostatic interactions in maintaining emulsion structure.

The digital inspection of o/w emulsions via polarized microscopy (Figure 2) corroborated the phase separation mechanisms inferred from the ESI calculations and droplet size estimations. The micrographs of CH/BNC1 (25:75) and CH/BNC1 (50:50) revealed similar heterogeneous dispersions composed of microsized droplets. In contrast, CH/BNC2 (25:75) and CH/BNC2 (50:50) revealed distinct phase separation phenomena, which aligns with the detected shear-thinning behaviors. CH/BNC2 (25:75) o/w emulsions showed partial coalescence at 7 d, with some droplets merging into larger ones while smaller droplets remained dispersed in the surrounding medium. The most pronounced phase separation was observed in CH/BNC2 (50:50) o/w emulsions, where multiple droplets aggregated while maintaining the structural integrity of the initial droplets. This phenomenon was attributed to flocculation [22], which also contributed to the erratic viscosity measurements. Overall, the excess CH content in both o/w emulsions promoted coalescence, leading to the formation of larger droplets. Notably, in the polarized micrographs of CH/BNC2 (75:25) o/w emulsions, the darker regions comprised densely packed small droplets, indicating gravitational separation that further facilitated sedimentation. These observations align with findings by Zhang et al., 2022 [32], who reported a significant reduction in droplet size in BC/carboxymethyl CH o/w emulsions as BC concentration increased (0–0.2 wt%). Additionally, the microparticle coverage of the droplet surface could be influenced by the molecular weight of CH used in microcrystalline cellulose/CH-dodecane oil o/w emulsions, leading to increased overall droplet size, as illustrated in optical micrographs by Ahsan et al., 2020 [31].

### 2.4. Effect of Environmental Stressors on Emulsions Stability

Numerous studies have established that emulsions exhibit both kinetic (resistance to changes in droplet size distribution over time) and thermodynamic stability (determines whether phase separation is inevitable); however, they are inherently susceptible to destabilization under unfavorable environmental conditions. Given that Pickering emulsions rely on the adsorption of solid particles at the oil-water interface to stabilize the dispersed phase, external factors such as pH fluctuations and ionic strength can significantly influence their stability [37].

To assess the robustness of CH/BNC-based emulsions, the impact of environmental stressors—specifically pH and ionic strength—on the ESI containing 0.5% wt. CH/BNC1 (25:75) complex was investigated. Among all tested o/w CH/BNC Pickering emulsions, CH/BNC1 (25:75) exhibited the highest emulsion stability, with an ESI being around 100% after 3-d of storage. Limited creaming and phase separation were observed even after 7-d of storage, suggesting that the CH/BNC1 (25:75) complex provided an effective interfacial barrier against coalescence and Ostwald ripening. The observed stability can be explained by the charge modulation and structural reinforcement imparted by the CH/BNC complex. CH, a cationic biopolymer, contributes electrostatic repulsion, preventing droplet aggregation, while BNC1 forms a rigid network that enhances viscosity and reduces droplet mobility.

**Figure 5:**
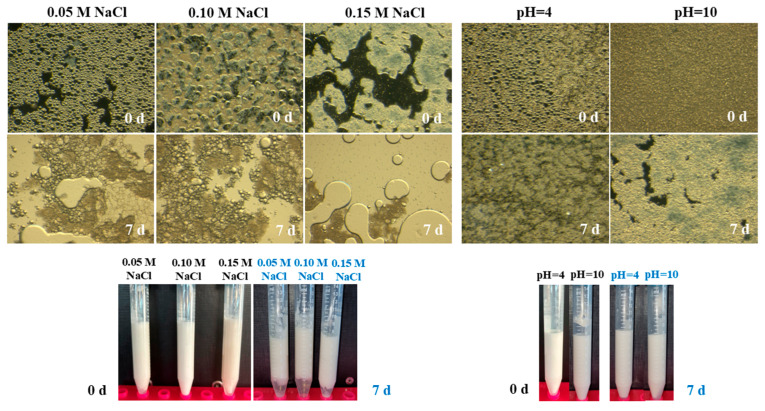
Microscopic (**top panel**) and visual evaluation (**bottom panel**) of complex o/w Pickering emulsions (20 *v*/*v* sunflower oil and 0.5 wt. total emulsifier) using complexes of chitosan (CH) and BC after H_2_SO_4_ treatment (BNC1) at a ratio of CH/BNC1_25:75_, under various pH values (4–10) and ionic strength (0.05–0.15 M NaCl), at the day of preparation (0 d) and after 7 d of storage at ambient temperature.

The stability of the emulsions after pH adjustment to 4 and 10 (Figure 5) was remarkable, with an ESI of 100%. Storage under ambient conditions had minimal impact on stability, as evidenced by the high ESI values recorded after 7 d—91% and 90% for emulsions prepared at pH 4 and 10, respectively. Notably, emulsions formulated under acidic conditions exhibited slightly higher stability after storage, likely due to enhanced interactions between positively charged CH and negatively charged bacterial BNC under acidic conditions, which contributed to a robust interfacial layer. The emulsions displayed moderate phase separation, with a droplet-rich cream layer at the top and a slightly turbid serum phase at the bottom, very similar to those prepared at neutral pH. These findings contrast with results reported by Zhang et al., 2022 [32] where BC/CH-caseinate-stabilized o/w emulsions at pH 3 exhibited immediate phase separation, leading to oiling-off, whereas emulsions prepared at pH 9.6 (with BC concentrations > 0.1 wt%) demonstrated the highest thermodynamic and kinetic stability during storage, attributed to depletion stabilization effects. The size and surface charge distributions of the emulsions varied significantly under different pH conditions. At acidic pH, emulsions exhibited a bimodal droplet size distribution, initially measured at 1.4 μm/8.3 μm, which increased slightly to 1.6 μm/19.4 μm after 7 d of storage. The emulsions also displayed low ζ-potential values (5.37 mV to 11.6 mV), indicating mild electrostatic repulsion. The enhanced protonation of CH under acidic conditions likely led to stronger electrostatic interactions with BNC, forming a more cohesive interface that supported emulsion stability. However, the viscous nature of CH may have contributed to limited coalescence, resulting in a moderate increase in droplet size over time. Conversely, emulsions prepared at pH 10 exhibited a more uniform and smaller droplet size distribution (initially 116 nm/598 nm, shifting slightly to 140 nm/534 nm after 7 d). The emulsions also displayed strongly negative ζ-potential values at both time points (−56.4 mV initially, decreasing to −24.7 mV after storage). At alkaline pH, CH remained largely neutral rather than protonated, while BNC particles retained their negative charge due to sulfate groups introduced during H_2_SO_4_ hydrolysis. The strong negative ζ-potential implied that BNC dominated, promoting stability through electrostatic repulsion, yet with no important aggregation phenomena. The work of Zhai XiChuan et al., 2018 [15] on o/w emulsions containing 0.05 wt% BNC nanofibers and 15% (*v*/*v*) peanut oil demonstrated that emulsions prepared at lower pH values (3 and 5) underwent coalescence due to reduced net charge and weakened repulsive interactions between droplets. In contrast, emulsions at higher pH values (7, 9, and 11) exhibited enhanced stability due to stronger electrostatic interactions preventing droplet fusion.

Microscopic analysis using polarized light microscopy (Figure 5) confirmed minimal destabilization during the 7-day storage. The observed droplet distributions and their densely packed configuration were consistent with the droplet size variations seen under acidic and alkaline conditions. At pH 3, protonation-induced coalescence (moderate) led to the merging of smaller droplets into larger ones, reinforcing previous observations of moderate instability under acidic conditions. These findings align with those of [38], whose work developed physically stable double-layered emulsions using complexes of BC nanofibers/soy PI/CH to encapsulate fat-soluble bioactive compounds such as curcumin. It was demonstrated that emulsions remained stable at pH 4 over a 4-day storage but exhibited significant creaming at pH 3, 6, and 8. While emulsions at pH 10 maintained visible stability, flocculation phenomena were observed, further supporting the role of pH-dependent electrostatic interactions in emulsion stabilization.

The ionic strength studies at different NaCl concentrations (0.05, 0.1, and 0.15 M) were subsequently conducted at pH 10 to simulate environmental conditions under which PI is soluble and thus could be sequentially enclosed. The initial ESI, close to 100% immediately after preparation across all NaCl concentrations, indicated effective stabilization. However, all tested NaCl concentrations induced moderate destabilization over storage. The ESI values ranged between 80–82%, accompanied by distinct phase separation phenomena. Notably, excessive NaCl (0.15 M) significantly reduced electrostatic repulsion, leading to droplet coalescence over time (ESI: 82%). Gravitational phase separation was also evident, with droplets merging, breaking, and ultimately sedimenting. At 0.1 M NaCl, droplets exhibited greater stability with minimal size increase during storage (0-d: 357 nm to 7.9 μm; 7-d: 429 nm to 7.5 μm), also accompanied by the most negative net charge (−35.2 to −13.4 mV), suggesting an optimal balance of electrostatic repulsion and steric stabilization. In contrast, 0.15 M NaCl induced the formation of larger microsized droplets over time (313–348 nm, 7.8–8.2 μm), indicating coalescence due to diminished electrostatic stabilization. The notable shift in ζ-potential values (−30.7 to 4.13 mV) during storage suggested excessive charge screening, facilitating droplet coalescence and lowering ESI. High NaCl concentrations can lead to coalescence and the breakdown of the gel-like network in emulsions due to the disruption of electrostatic interactions and destabilization of the interfacial structure. Moreover, at high NaCl levels, Na^+^ ions may bridge the negatively charged sites on droplet surfaces, which are mainly composed of BNC1, further promoting aggregation and rapid phase separation. The lowest ionic strength (0.05 M NaCl) maintained emulsion stability but resulted in weaker electrostatic repulsion compared to 0.1 M NaCl, as reflected in the net charge (−15.5 to −16.1 mV). This led to slight instability over time.

The micrographs received by polarized microscopy revealed stability mechanisms consistent with size distribution studies (DLS/ELS). Emulsions with the lowest NaCl concentration formed flocs or larger droplets in a rather heterogeneous system (Figure 5). In contrast, 0.15 M NaCl promoted partial droplet collapse and coalescence regions (Figure 5). These results suggest that 0.1 M NaCl provided the optimal ionic strength for emulsion stability in o/w CH/BNC1 (25:75) emulsions, achieving a balance between droplet stability, electrostatic repulsion, and size distribution.

The flow rheological curves indicated that the emulsions’ non-Newtonian behavior resulted in a shear-thinning feature across pH 3, 7, and 10 (Figure 3c). Higher apparent viscosity was detected in the case of basic media used for the o/w formulation. The Pickering stabilization effect from BNCI might be sufficient to maintain the emulsion’s stability and allow for shear-thinning behavior. Increased viscosity was also detected after the salt addition (0.05M) as a consequence of the amphiphilic properties of the BNC1 originated by the crystalline structure, hydroxyl and carboxyl groups, and strong hydrogen bonds in the polymer chains [39]. The salt additions of 0.1M and 0.15M potentially disrupted the electrostatic interactions that are crucial for stabilization, leading to unreliable rheological metrics. Pinto et al., 2024 [25] extensively discussed the formation of low o/w Pickering emulsions BNC using either nanocrystals or nanofibrils. In the absence of salt, emulsion stability was enhanced by higher BC concentrations (1%), but long-term stability of the emulsions was determined by the concentration of salt at lower BC concentrations (0.5%).

### 2.5. Dynamic Shear Rheology of CH/BNC1(25:75)-Based Emulsions

**Figure 6:**
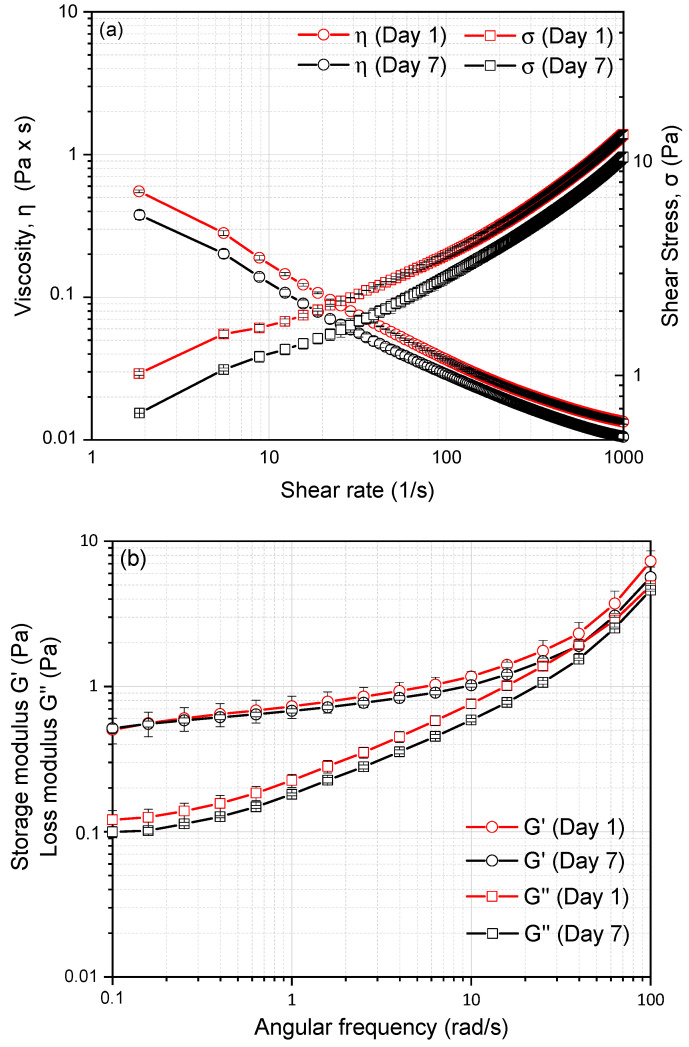
Flow behavior of CH/BNC1_25:75_-based Pickering emulsions stored for 7 days: (**a**) apparent viscosity (η), shear stress (σ) vs. shear rate ($\dot{\gamma }$) respectively and viscoelastic properties: (**b**) Storage modulus (G′) and loss modulus (G″). Data are presented as mean ± standard deviation from two independent experimental replicates.

Pickering emulsions stabilized with CH/BNC1 (25:75) complexes were further investigated by measuring the flow behavior and viscoelastic properties. The apparent viscosity was plotted as a function of shear rate, revealing that the viscosity of the samples decreases with an increase in shear rate. This behavior indicates a typical non-Newtonian, pseudoplastic fluid with time-dependent shear-thinning properties over a wide range of shear rates (1–1000 S^−1^) (Figure 6). Moreover, the apparent viscosity of the emulsion decreased slightly over storage time (7 days) in a consistent pattern, suggesting minor structural changes without significant destabilization, while its role in limiting droplet movement proves more effective in stabilizing the emulsion than controlling droplet size alone. This method was used as a tool for the stability mechanism of the system. Moreover, from the plot of shear stress as a function of shear rate (0.1–1000 s^−1^) (Figure 6a), we used the rheological Power law model to further elucidate the pseudo-plastic (non-Newtonian) behavior. The rheological parameters obtained from the Power-law model and presented in Table 3 demonstrated an excellent fit to the experimental data. Additionally, the flow behavior index (m) of all samples was in the range of 0–1, indicating CH self-assembled particles stabilized Pickering emulsion exhibited shear-thinning (pseudoplastic) behavior in accordance with previous studies [40]. Moreover, the lower κ value indicates that the emulsion has better fluidity under the action of an external force, reflecting slight instability during the 7-day storage period.

The viscoelastic properties of emulsions play a critical role in determining their physical properties, including appearance and stability, making rheological analysis a valuable tool for elucidating the colloidal structure of emulsion-based food systems [41,42]. The relationship between G′ and G″ as a function of angular frequency is presented in Figure 6b. Throughout the measured frequency range, the elastic modulus G′ is always greater than the loss modulus G″ for all samples, and they do not intersect each other, indicating a solid-like nature. This phenomenon may be attributed to the formation of a three-dimensional network through droplet interactions in the Pickering emulsions and presents the characteristics of a typical weak gel [43,44,45]. The solid-dominant behavior may result from particle adsorption at oil-water interfaces and the formation of a percolated lipid droplet network, enabling the rearrangement of colloidal particles to form a strong network architecture that effectively acts as a particle stabilizer [42].

### 2.6. Evaluation of CH/BNC1 (25:75) Emulsifiers as Carriers for Renewable Proteins

Water-soluble proteins, such as PI derived from sunflower seeds (*Helianthus annuus*), exhibit limited environmental stability when exposed to various stimuli, including pH fluctuations, temperature changes, and ionic strength variations [46].

As a result, their application as emulsifiers in food-grade emulsions often leads to phase separation (due to flocculation and coalescence phenomena) [47]. To address these functional limitations, researchers have explored both covalent and non-covalent interactions between plant proteins and polysaccharides. These protein-polysaccharide complexes can be formed without the need for enzymatic or chemical modifications while simultaneously enhancing solubility and emulsifying capacity [48].

**Figure 7:**
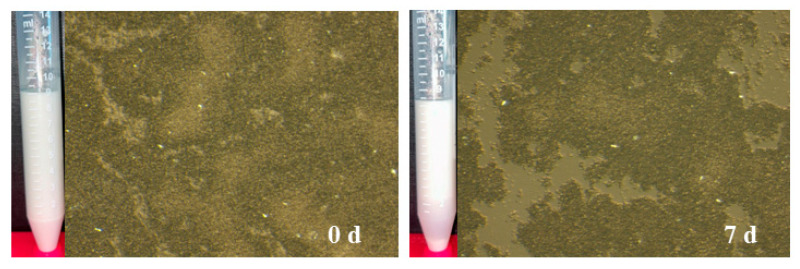
Microscopic and visual evaluation of complex o/w Pickering emulsions (20 *v*/*v* sunflower oil and 0.5 wt. total emulsifier) using complexes of chitosan (CH) and BC after H_2_SO_4_ treatment (BNC1) at a ratio of CH/BNC1_25:75_, combined with protein isolates extracted from sunflower seed at the day of preparation (0 d) and after 7 d of storage at ambient temperature.

A preliminary evaluation of CH/BNC1 (25:75) emulsifier as nanocarrier for SFS PI was performed (Table 1, Figure 7). The freshly prepared emulsions demonstrated an exceptional ESI of 100% on the day of formulation. However, phase separation phenomena emerged during storage, resulting in sedimentation by day 7. The emulsions developed a distinct three-layered system. The upper and middle layers, differing in opacity, consisted of varying droplet sizes, whereas the bottom layer contained sedimented protein/CH/BNC1 complexes that failed to establish a stable interfacial layer around the SO droplets. Over the storage period, droplet size decreased from 90 nm and 1.4 μm to 258 nm, displaying significant heterogeneity (PDI ~ 0.4), as corroborated by polarized microscopy images. Concurrently, the surface charge of the emulsions increased from 9.72 mV to 32.8 mV, indicating the progressive formation of compacted structures. The non-covalent interactions between the negatively charged PI (ζ = −41.1 mV at pH = 10) [49] and positively charged polysaccharide CH/BNC1 (25:75) complexes created closed-packed coacervates. Similar behavior has been observed by [50] who demonstrated that soybean PI, when combined with CH (cationic polysaccharide) or anionic carboxymethyl cellulose, forms stable coacervates with promising applications as emulsifiers. Overall, the preliminary results demonstrated a promising CH/BNC1 nanocarrier that could potentially be utilized not only for protein complexation but also for the incorporation of other hydrophobic or sensitive substances, such as essential oils, for future application in the food industry [51].

## 3. Conclusions

This study highlighted the potential of CH/BNC nanocomplexes—particularly at a 25:75 ratio—as effective, natural stabilizers for o/w Pickering emulsions. These hybrid systems significantly enhanced emulsion stability by improving viscosity, maintaining uniform droplet distribution, and reinforcing interfacial barriers. The emulsions exhibited strong resistance to environmental stressors, including pH variation and ionic strength, with optimal performance at 0.1 M NaCl. Rheological analysis confirmed the long-term viscoelastic stability of the CH/BNC1 (25:75)-based emulsions. While the incorporation of SFS PI introduced phase separation over time, this behavior underscores the complexity of protein–biopolymer interactions and their influence on emulsion structure. Overall, this work offers a sustainable platform for designing multifunctional emulsifier systems with promising applications in food and nutraceutical formulations.

## 4. Materials and Methods

### 4.1. Materials

CH (MW of 162 kDa) and acetic acid (factor: 0.998) were purchased from Sigma-Aldrich (Athens, Greece). The fermentation to produce BC was carried out with the bacterial strain *Komagataeibacter rhaeticus* UNIWA AAK2 at 30 °C, using 10% (*v*/*v*) of pre-culture, for 7 days using a static incubator. The fermentation media consisted of (in g/L) glucose 20, yeast extract 5, peptone 5, citric acid 1.15, and Na_2_HPO_4_ 2.7. BC purification, and hydrolysis using H_2_SO_4_, and H_2_SO_4_-HCl mixture to obtain bacterial nanocellulose (BNC1 for BC obtained from H_2_SO_4_-assisted hydrolysis and BNC2 for H_2_SO_4_-HCl-assisted hydrolysis) have been described in detail by Efthymiou, Tsouko, Pateraki, et al., (2022) [52]. The production of protein isolates from sunflower seed (SFS PI) was performed using alkaline treatment for protein solubilization (pH was fixed to 10 using 5 M NaOH) followed by acidification with 5 M HCl to achieve protein precipitation at the isoelectric point, as presented by Vardaxi et al., 2024 [49]. Sunflower oil (SO) was purchased from a local market (Athens, Greece). All aqueous solutions were prepared with deionized water.

### 4.2. Production of Simple-Emulsifier (Parent) O/W Pickering Emulsions

Emulsions were formulated using CH solution, BC dispersions, BNC1 dispersions, and BNC2 dispersions. To specify, CH was dissolved in 2% *w*/*v* acetic acid under magnetic stirring. BC, BNC1, and BNC2 were dispersed in deionized water and left overnight under magnetic stirring to allow for swelling. The concentration of each emulsifier was set at 0.5 *w*/*v*. Then, each of the aforementioned solution/dispersion was homogenized with SO at a volumetric ratio of 5:1, using high shear homogenizer (Ultraturrax homogenizer-IKA, t 25 basic, Staufen im Breisgau, Germany) (2 min at 14,000 rpm) at ambient temperature followed by ultrasonication (50 kHz, 500 W, Sonica 3300ETH-S3, Soltec, Milan, Italy) for 2 min at 40% amplitude in pulsed mode (2 s on/2 s off) [23]. After the homogenization and sonication treatment, the freshly prepared emulsions were stored for 7 days and placed under ambient conditions to investigate their stability.

### 4.3. Preparation of CH/BC-Based Complexes

To produce the complexes, CH solutions and BNC1, and BNC2 dispersions were first prepared separately as described in Section 2.2. Next, each BNC-based dispersion was mixed with the CH solution to create complex dispersions with total complex concentration of 0.5% *w*/*v*. The final concentration of complex dispersions has been determined based on emulsifier concentration in the formulation of complex-emulsifier emulsions. Complexes were formulated in varying ratios: CH_BNC1 (25/75) CH_BNC1 (50/50), CH_BNC1 (75/25), and CH_BNC2 (25/75) CH_BNC2 (50/50), CH_BNC2 (75/25). All complex dispersions were stored in ambient conditions for further experiments.

### 4.4. Production of Complex-Emulsifier O/W Pickering Emulsions

All the produced complexes were utilized for o/w Pickering emulsion preparations as described in Section 2.2. Briefly, the CH/BNCs complexes were prepared at different biopolymer mixing ratios and final total emulsifier concentration of 0.5% *w*/*v*. The complex emulsifiers were mixed with SO at a volumetric ratio of 5:1, then homogenized using high sheer homogenizer and sonicated for 2 min at 40% amplitude in pulsed mode. The produced complex-emulsifier o/w emulsions were stored under ambient conditions for further characterization.

### 4.5. Stability Evaluation of Complex-Emulsifier O/W Pickering Emulsions Under Different Values of pH and Ionic Strength

The effect of pH and ionic strength on o/w Pickering emulsions stabilized by selected CH/BNC complexes was examined. The pH stability test was performed by adjusting the pH of the selected CH/BNC dispersions to ca. 3, 7, and 10 (using 0.1 M HCl or 0.1 M NaOH) prior to the oil mixing and homogenization step. Similarly, the ionic strength stability test was performed by dispersing the selected CH/BNC complexes into 50 mM, 100 mM, and 150 mM NaCl prior to their involvement in the emulsification process.

### 4.6. Complexation Studies of O/W Emulsions with Protein

The optimal CH/BNC ratio containing 0.5% wt. emulsifiers was eventually evaluated as nanocarriers for SFS complexation. Aqueous dispersions of CH, BNC, and SFS (dissolved at pH 10.5 using 5 M NaOH) were prepared separately and allowed overnight to swell. SO (20% *v*/*v*) was added to the final emulsifier complex (0.5% *w*/*v*), and the mixture was homogenized, sonicated, and stored for 7 days in falcon tubes until further characterization.

### 4.7. Characterization of O/W Emulsions and Complexes

#### 4.7.1. Emulsion Stability Index (ESI)

The visual inspection of droplet movement was used to assess the emulsions’ resilience towards structural changes over 7 days of storage at ambient temperature and at specific time intervals. Due to their low density, the emulsion droplets tend to separate due to gravity and migrate to the top layer, forming an emulsion layer (also known as the cream layer), with the serum layer at the bottom [17]. The height of the emulsifying layer (H_e_) and the total height of emulsions (H_c_) were measured from t = 0 to t = 7 days in order to calculate their ESI using the following equation [23]:

(1) $$\mathrm{ESI}\;=\;\frac{H_{e}}{H_{C}}\times 100\%$$

#### 4.7.2. Particle Size and ζ-Potential Measurements

The particle size, size distribution, and surface charge of emulsions and complexes were characterized using dynamic and electrophoretic light scattering techniques (DLS/ELS). The DLS studies were conducted on an ALV/CGS-3 compact goniometer system (ALV GmbH, Langen, Germany) equipped with an ALV-5000/EPP multi-τ digital correlator and a 22 mW He–Ne laser (λ = 632.8 nm) as the light source. The freshly prepared emulsions were measured at different scattering angles (ranging from θ = 45° to θ = 145°). Five measurements lasting 30 s were obtained at each angle. The collected autocorrelation functions were analyzed using the cumulants method and the CONTIN algorithm. The size at the maximum of the distribution was identified as the emulsion droplets’ average hydrodynamic radius (R_h_). The fresh emulsions were diluted 1:10,000 to avoid multiple scattering effects. All experiments were performed in duplicates at 25 °C ± 5 °C.

The zeta potential was calculated using the Zetasizer Nano-ZS (Malvern Instruments Ltd., Malvern, UK). An average of 50 runs at a backscattering angle of θ = 173° was used to evaluate the ζ-potential. The emulsions were prepared with a similar approach as outlined for the DLS measurements [53]. The solution/dispersions were prepared and left overnight to equilibrate, and subsequently, the R_h_ and surface charges were determined as described above.

#### 4.7.3. Viscosity Measurements

A Brookfield DV-I PRIME (Middleboro, MA, USA) cone-and-plate digital viscometer was used to assess viscosity in bulk. Following torque equilibrium at a constant value, a viscosity measurement was made for a specific shear rate. The experiment was conducted at various shear rates, ranging from low to high, and its repeatability was verified by testing both low and high shear rates multiple times. The range of observable shear rate that was reported was the outcome of the instrument’s restrictions with regard to minimum and maximum measurable torque [54]. The required amount of the emulsions was added to the flat plate, and every test was run in duplicates and measured at 25 °C.

#### 4.7.4. Dynamic Shear Rheological Measurements

The rheological properties of the emulsions were examined immediately following preparation, while stability was assessed after a 7-day storage period at an ambient temperature of 25 °C. Measurements of the dynamic and steady shear rheological properties of samples were obtained using a stress-controlled rheometer (Discovery HR-3, TA Instruments, New Castle, DE, USA) equipped with a concentric cylinder geometry (30-mm cup diameter, 28-mm Bob diameter, 42-mm Bob length). The temperature was kept constant (25.0 °C) using a Peltier thermoelectric heat pump system. After positioning, the samples equilibrated for 2 min prior to rheological measurements, and all data presented as the average of triplicate measurements.

Dynamic frequency sweep was performed using a constant strain (LVR region), at a frequency of 0.1–100 rad/s. The dynamic storage (G′) and loss (G″) moduli were obtained.

Shear stress (τ in Pa), shear rate ($\dot{\gamma }$ in s^−1^), and apparent viscosity (η) were also obtained at a shear rate range from 1 to 1000 s^−1^ for samples. The relationship between τ and $\dot{\gamma }$ was described by the following power law model:

(2) $$\tau =\kappa \times {\dot{\gamma }}^{n}$$

where, K is the consistency coefficient (Pa·s^n^), and n is the flow behavior index (dimensionless). R^2^ is the fitting correlation coefficient of the equation. Moreover, the apparent viscosity (η), which is also modeled by a power-law dependence on shear rate was modelled as follows to obtain a prefactor (N) and a flow index (m).

(3) $$\eta \left(\dot{\gamma }\right)=N\times {\dot{\gamma }}^{-m}$$

#### 4.7.5. Microstructural Morphology

The crystalline morphology of emulsions was observed using a stereoscope equipped with a digital camera and polarized light (Leica MZ16, Microscope Service & Sales Ltd., Buckinghamshire, UK). Images were acquired using a 116× magnification.

## Figures and Tables

**Table 1:** Dispersion characteristics of neat biopolymers, CH/BNCs complexes, parent o/w Pickering emulsions, and complex o/w Pickering emulsions.

Compounds	Storage (d)	ζ-Potential (mV)	R_h_ (nm)	PDI
Biopolymers				
CH	-	54.1 ± 4.8	84 nm/859 nm/6.8 μm	0.52
BC	-	−16.6 ± 6.5	69 nm/584 nm/637 nm	0.50
BNC1	-	−34.7 ± 4.1	74 nm/471 nm	0.49
BNC2	-	−27.5 ± 3.8	60 nm/387 nm/3 μm	0.49
CH/BNC complexes				
CH/BNC1_25:75_	-	5.7 ± 2.3	228 nm/7.8 μm	0.46
CH/BNC1_50:50_	-	10.5 ± 3.9	1.6 μm/8.5 μm	0.29
CH/BNC1_75:25_	-	22.5 ± 2.9	1 μm/8.6 μm	0.43
CH/BNC2_25:75_	-	5.3 ± 1.3	1.2 μm/8.1 μm	0.39
CH/BNC2_50:50_	-	11.6 ± 5.2	1.9 μm/8.6 μm	0.36
CH/BNC2_25:75_	-	22.1 ± 3.5	56 nm/1.41 μm/8 μm	0.33
Parent o/w emulsions				
CH	0	4.9 ± 1.3	134 nm/7.5 μm	0.48
	7	10.7 ± 2.8	211 nm/8.1 μm	0.47
BC	0	−8.60 ± 3.2	147 nm/7.8 μm	0.42
	7	−29.1 ± 5.7	260 nm/7.6 μm	0.37
BNC1	0	−12.4 ± 3.1	81 nm/192 nm/8 μm	0.43
	7	−27.2 ± 3.9	120 nm/1.1 μm/8.1 μm	0.36
BNC2	0	−21.8 ± 2.7	89 nm/156 nm/8 μm	0.33
	7	−27.3 ± 2.5	2.3 μm/8.5 μm	0.34
Complex o/w Pickering emulsions				
CH/BNC1_25:75_	0	5.9 ± 1.3	75 nm/8.4 μm	0.38
	7	5.7 ± 1.3	228 nm/7.8 μm	0.46
CH/BNC1_50:50_	0	11.7 ± 4.0	164 nm/1.4 μm/8.5 μm	0.30
	7	10.5 ± 3.9	1.6 μm/8.5 μm	0.29
CH/BNC1_75:25_	0	21.4 ± 3.4	1.9 μm/8.3 μm	0.30
	7	22.5 ± 2.9	1 μm/8.6 μm	0.43
CH/BNC2_25:75_	0	5.2 ± 3.6	1.9 μm/8.3 μm	0.31
	7	5.3 ± 2.3	1.2 μm/8.1 μm	0.39
CH/BNC2_50:50_	0	−1.3 ± 0.1	32 nm/354 nm/1.8 μm	0.46
	7	11.6 ± 4.2	1.9 μm/8.6 μm	0.36
CH/BNC2_75:25_	0	13.5 ± 3.6	90 nm/1 μm/8.3 μm	0.52
	7	22.1 ± 3.5	56 nm/1.4 μm/8 μm	0.33
Effect of pH and ionic strength on CH/BNC1_25:75_ based emulsions				
CH/BNC1_25:75_—pH = 4	0	5.4 ± 2.3	1.4 μm/8.3 μm	0.43
	7	11.6 ± 3.4	1.6 μm/19.4 μm	0.37
CH/BNC1_25:75_—pH = 10	0	−56.4 ± 6.7	116 nm/598 nm	0.51
	7	−24.7 ± 3.1	140 nm/534 nm	0.49
CH/BNC1_25:75_—0.05 M NaCl	0	−15.5 ± 5.9	497 nm/7.8 μm	0.32
	7	−16.1 ± 3.4	406 nm	0.48
CH/BNC1_25:75_—0.1 M NaCl	0	−35.2 ± 8.7	357 nm/7.9 μm	0.32
	7	−13.4 ± 4.7	429 nm/7.5 μm	0.49
CH/BNC1_25:75_—0.15 M NaCl	0	−30.7 ± 4.1	313 nm/7.8 μm	0.36
	7	−4.1 ± 2.1	348 nm/8.2 μm	0.41
o/w emulsions stabilized with CH/BNC1_25:75_ and SFS PI				
CH/BNC1_25:75_—SFS	0	9.7 ± 3.7	90 nm/1.4 μm	0.49
	7	32.8 ± 8.5	258 nm	0.48

R_h_: hydrodynamic radius, PDI: polydispersity index, CH: chitosan, BNC1: bacterial nanocellulose obtained from H_2_SO_4_-assisted hydrolysis, BNC2: bacterial nanocellulose obtained from H_2_SO_4_-HCl-assisted hydrolysis, SFS PI: protein isolates from sunflower seed. Data are presented as mean ± standard deviation from two independent experimental replicates. Note: The presence of multiple R_h_ values in most of the examined samples indicates heterogeneous particle size distributions, as revealed by DLS measurements.

**Table 2:** Values of the prefactor (N) and flow index (m) as obtained from power-law dependence on shear rate (Equation (3)).

Sample	N	m
BNC2	525	0.63
CH/BNC1_25:75_	427	0.61
CH/BNC1_50:50_	295	0.45
CH/BNC1_75:25_	240	0.34
CH/BNC2_25:75_	1778	0.82
CH/BNC2_25:75_	51	0.035

**Table 3:** Determination of rheological parameters of Pickering emulsions stabilized with CH/BNC1(25:75) complexes using Power-law model (Equation (2)).

Storage (Days)	k	n	R^2^
1	0.211	0.597	0.993
7	0.052	0.586	0.995

## Data Availability

The original contributions presented in this study are included in the article. Further inquiries can be directed to the corresponding author.
